# Arthroscopic Excision of a Solitary Intra-articular Osteochondroma Manifesting as a Loose Body: A Presentation of an Extremely Rare Medical Condition

**DOI:** 10.7759/cureus.66083

**Published:** 2024-08-03

**Authors:** Sai Kulkarni, Vijayalaxmi S Patil, Sandeep Naik, Yogita Bhansali

**Affiliations:** 1 Pathology, Shri B. M. Patil Medical College, Hospital and Research Centre, Bijapur Lingayat District Education (Deemed to be University), Vijayapura, IND; 2 Pathology, Bijapur Lingayat District Education (Deemed To Be University), Vijayapura, IND; 3 Orthopedic Surgery, Shri B. M. Patil Medical College, Hospital and Research Centre, Bijapur Lingayat District Education (Deemed to be University), Vijayapura, IND

**Keywords:** the knee joint, arthroscopy, elder, intra-articular loose body, solitary osteochondroma

## Abstract

Osteochondroma typically has extra-articular growths at the metaphysis. Intra-articular osteochondroma is extremely uncommon. We report a case of a 55-year-old woman who had been experiencing right knee pain for the past 12 months. An arthroscopy revealed a medial meniscus tear with a loose body in the right knee. It was removed arthroscopically. Histopathology identified it as an osteochondroma. Therefore, intra-articular osteochondroma can be regarded as an uncommon cause of loose bodies in adult patients.

## Introduction

Osteochondroma/exostosis is the most common benign bone tumor. It occurs in growing bones and comprises mature bone covered with a cartilaginous cap [1]. It frequently arises from the metaphysis, where it attaches with a stalk and grows toward the diaphysis [2]. It can be solitary or multiple. They are believed to have started as little cartilaginous nodules within the periosteum and are most likely to be developmental abnormalities rather than actual tumors [3]. These lesions are usually asymptomatic and are located near the proximal end of the femur and humerus, positioned extra-articularly [4]. The biological potential of intra-articular osteochondromas affecting the knee joint is poorly understood and frequently misdiagnosed. These lesions, which can be rather large, have histologic traits that point to a malignant process, such as atypia of individual chondrocytes and hypercellularity of the cartilaginous component. Soft tissue tumors frequently develop in close proximity to joints without attaching to the bone. Furthermore, some of these tumors continue to expand even after the skeleton has fully developed [4]. Here, we provide an instance of intra-articular osteochondroma in a 55-year-old female patient complaining of pain and discomfort in the right knee joint, which appeared as a single body during arthroscopy.

## Case presentation

A 55-year-old, well-nourished female had pain and discomfort in the right knee. She had a history of falls four years back. Since then, she developed pain in the right knee, which was insidious in onset, gradually progressive, continuous sharp type, and nonradiating. The pain had aggravated in the last year, and the patient was having difficulty climbing stairs. The patient is a known case of hypertension and has been on antihypertensive medications for the last five years. There was no significant family history. On inspection, there was a diffuse swelling over the right knee. The skin over it appeared normal. No scars, discharging sinus, and dilated veins were noted. All the inspection findings were confirmed by palpation. Tenderness was noted over the right medial joint line. Distal pulse and distal movements were present, and the neurovascular deficit was not present. The range of motion of the right and left knee joints was 0 to 120 degrees, and the right knee showed valgus stress.

Investigations

**Figure 1:**
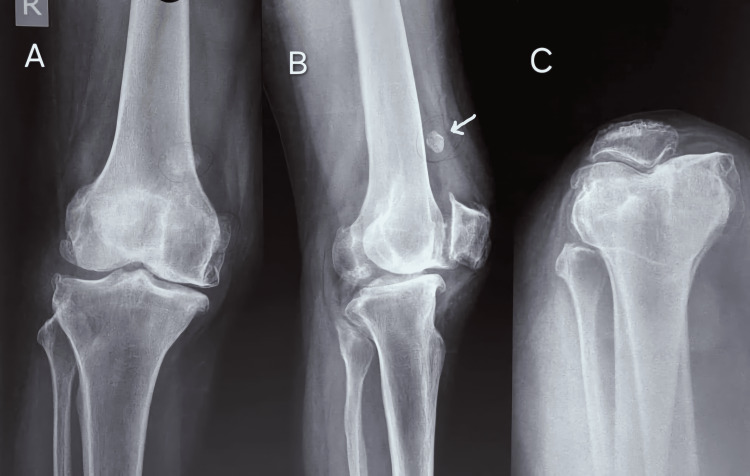
Anteroposterior (A,C) and lateral (B) views of the patient’s knee on a plain radiograph showing a radio-opaque shadow of an intra-articular loose body (white arrow)

Clinical indicators, plain radiography, and MRI were used to diagnose the condition. Anteroposterior and lateral plain radiographs showed an intra-articular loose body (Figures 1A-1C).

**Figure 2:**
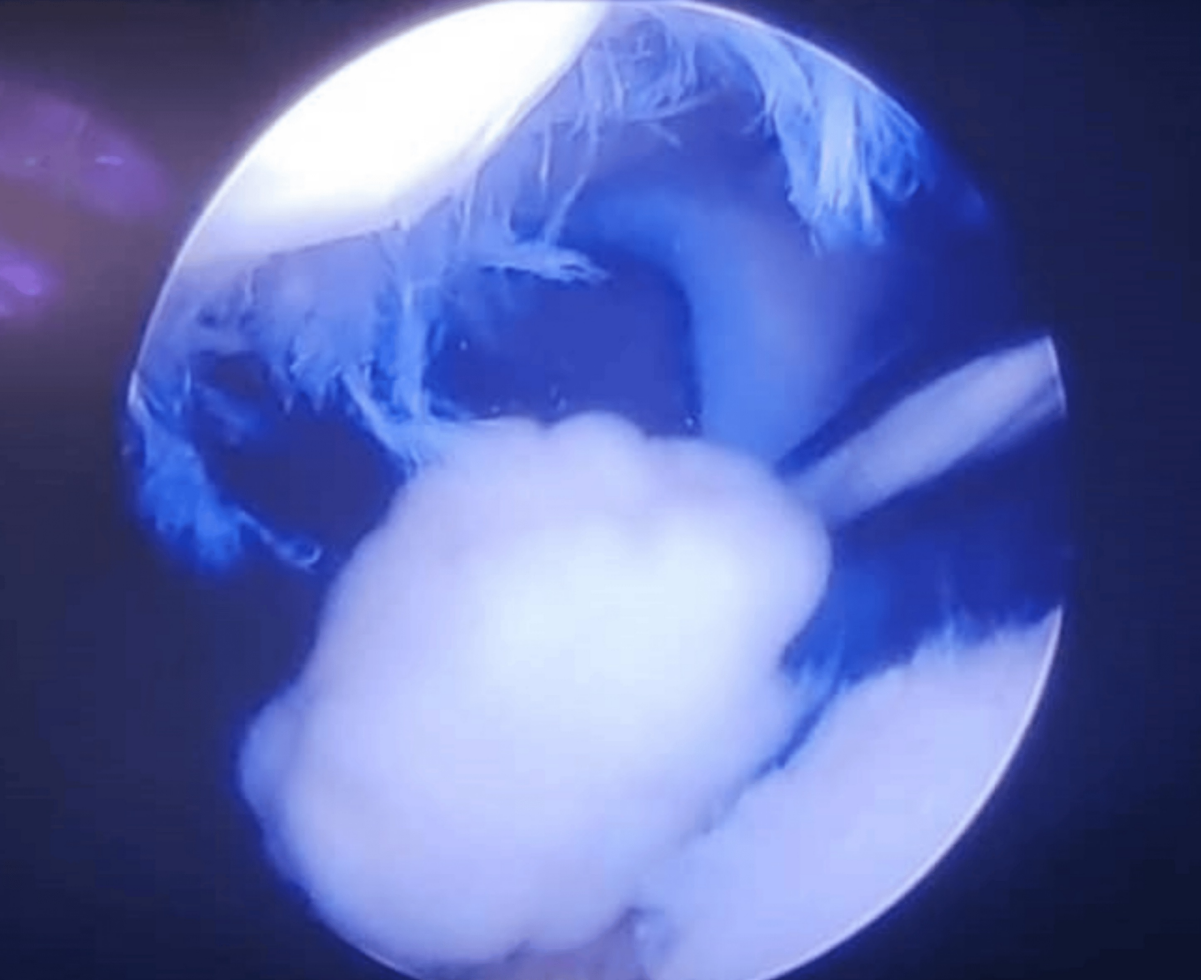
Arthroscopic view of the knee joint showing solitary loose body

MRI scan revealed an intra-articular mass (hyperintense to muscle) in the intercondylar notch. The patient underwent arthroscopic meniscus stabilization and removal of loose body, which was sent for histopathological evaluation. Arthroscopy revealed a right knee medial meniscus degenerative tear with a floating loose body about 2 cm × 2 cm in size with no evidence of connectivity with bone (Figure 2).

**Figure 3:**
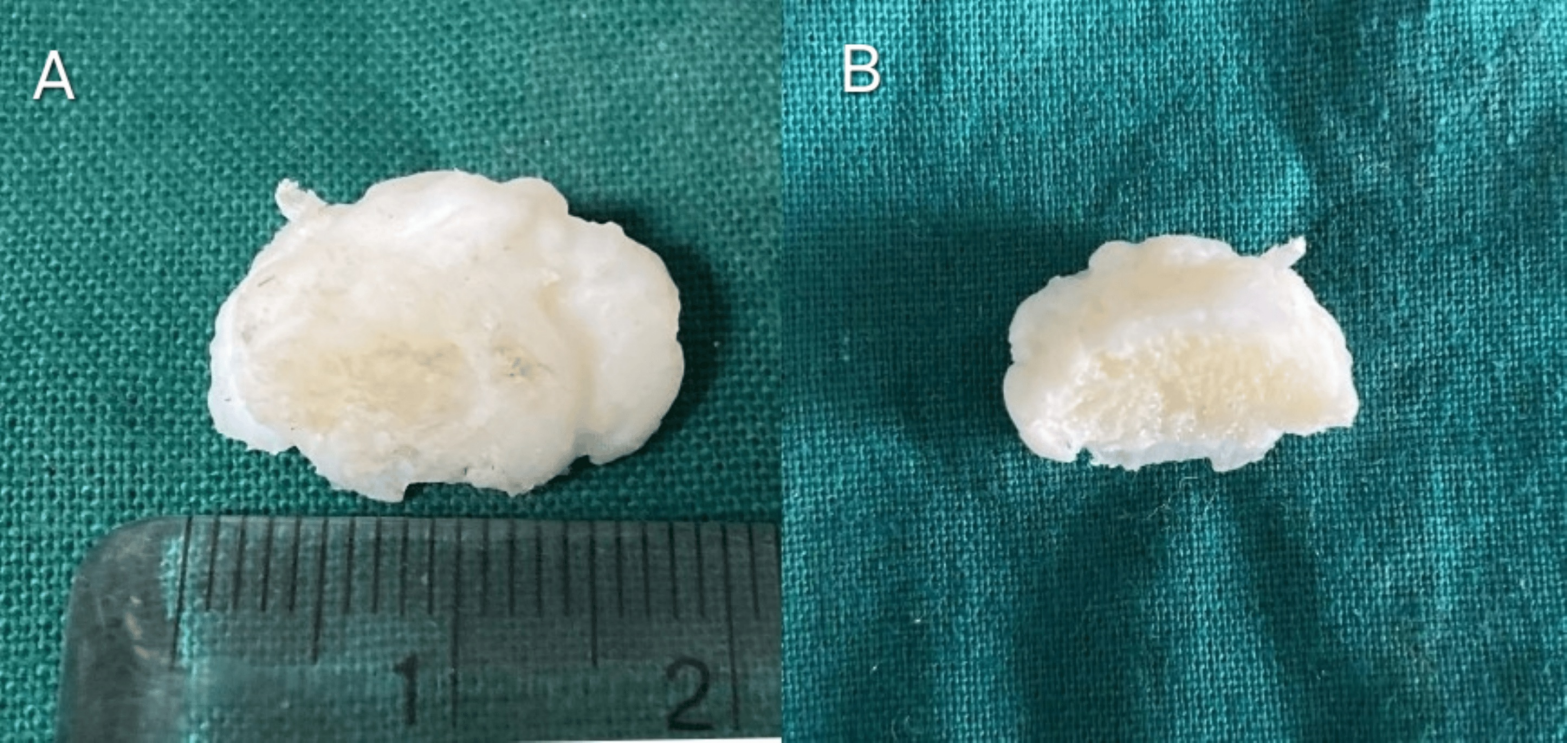
Gross specimen of the lesion showing globular pale white glistening tissue (A). Cut section showing central pale-yellow area surrounded by glistening capsule (B)

Grossly, it was a globular pale white glistening tissue measuring 2 cm × 2 cm in size. The cut section was hard and showed a central pale-yellow area surrounded by a thin pale-white glistening zone (Figures 3A, 3B).

**Figure 4:**
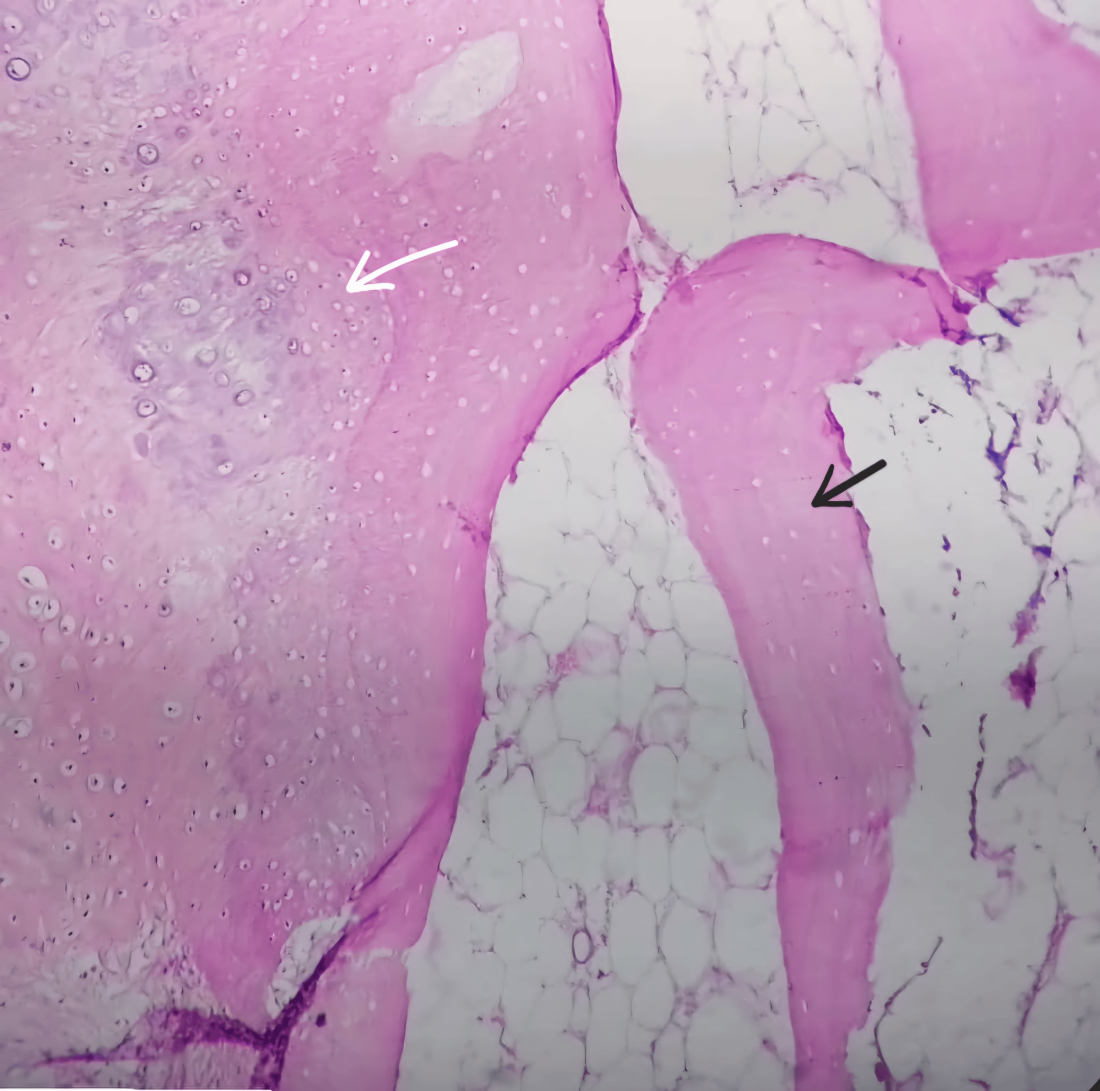
Photomicrograph showing cartilaginous cap (white arrow) over bony trabeculae (black arrow)

Microscopic examination revealed the presence of mature bony trabeculae enclosing fatty marrow spaces, all of which were encapsulated by a hyaline cartilaginous membrane. There was no evidence of mitotic figures/atypia in the chondrocytes (Figure 4). Thus, the diagnosis of osteochondroma was made.

Outcome and follow-up

Following the arthroscopic removal of the benign tumor, the patient experienced complete relief from her symptoms. Within a month of the surgery, she resumed her routine activities without any subsequent complaints.

## Discussion

Osteochondromas are the most common benign bone tumors, typically found near the growth plates of long bones in children and young adults [1]. They are believed to originate from abnormal growth of the epiphyseal growth plate, where a portion of the cartilage becomes displaced and continues to grow independently [2]. On imaging, osteochondromas usually appear as an exostosis with a continuous medullary cavity with the underlying bone. Radiographs often reveal the continuity of the cortex and medullary space between the osteochondroma and the parent bone, which is crucial for diagnosis. MRI is particularly valuable for evaluating the cartilaginous cap and distinguishing osteochondromas from malignant lesions such as chondrosarcoma, especially when symptoms are present or when the cartilaginous cap is thickened [3].

Morphologically, osteochondromas vary in size and shape, ranging from a few millimeters to several centimeters. Sessile osteochondromas are broad-based outgrowths, while pedunculated types have a stalk-like base [4]. Histologically, the tumor's cap consists of a cartilaginous layer resembling normal epiphyseal cartilage, which can undergo endochondral ossification. The underlying bone of an osteochondroma is continuous with the cortical and cancellous bone of the host bone, which is a key diagnostic feature [4].

Chouliaras et al. [1] described a 32-year-old woman showing a sessile bony growth arising from the superolateral aspect of the distal femur, as revealed by MRI. Arthroscopic excision provided symptomatic relief. Histopathological examination confirmed the diagnosis, showing a benign cartilage cap overlying bone without signs of atypia or malignancy.

Morey et al. [2] reported that a 16-year-old patient experienced knee discomfort and clicking. The patient was diagnosed with an intra-articular osteochondroma via arthroscopy. The morphological examination revealed a typical cartilage cap seen in osteochondromas, which was continuous with the underlying bone.

Mohanen et al. [4] and Kong et al. [5] discussed rare presentations of intra-articular osteochondromas manifesting as loose bodies within the joints. Patients exhibited joint pain and mechanical symptoms, such as locking or restricted motion. Morphological assessment confirmed the presence of osteochondromas, characterized by a cartilage cap and a bony stalk continuous with the host bone. Histopathology was crucial in differentiating osteochondromas from other conditions, such as synovial chondromatosis or secondary chondrosarcoma.

In this case, a 32-year-old female presented with persistent knee pain and mechanical discomfort for three months. MRI revealed a sessile bony growth at the superolateral aspect of the distal femur. This presentation is atypical, as osteochondromas generally affect younger individuals and are usually extra-articular. The symptoms and imaging findings were consistent with previous reports of intra-articular osteochondromas, which tend to cause more pronounced clinical symptoms than their extra-articular counterparts.

Accurate diagnosis of intra-articular osteochondromas relies heavily on imaging studies. While plain radiographs can identify extra-articular osteochondromas, they may not provide sufficient details for intra-articular lesions. MRI is crucial to assessing the exact size, location, and impact on surrounding joint structures [5]. Histopathological examination is indispensable in the evaluation of osteochondromas. It provides definitive diagnostic confirmation, differentiates benign tumors from malignant ones, and offers insights into the lesion's potential for malignant transformation [3].

The management of symptomatic osteochondromas typically involves surgical excision. Arthroscopic resection is favored for intra-articular osteochondromas due to its minimally invasive nature, reduced postoperative pain, superior cosmetic outcomes, and faster recovery compared to open surgery. Studies by Chouliaras et al. [1] and Kim et al. [3] reported successful outcomes with arthroscopic excision of intra-articular osteochondromas, emphasizing the procedure's efficacy and safety.

## Conclusions

Osteochondromas, while common and generally benign, can present with significant clinical symptoms when located intra-articularly. Recognizing the pathological and morphological features of these tumors is crucial for accurate diagnosis and appropriate management. The unique presentation and location of the osteochondroma in our patient underscores the importance of considering this entity in the differential diagnosis of joint-related symptoms and highlights the effectiveness of arthroscopic intervention in such cases.
